# Seasonal dynamics of honey bee pathogens and *Varroa destructor* infestation in South Korea: results from a National Active Surveillance Program

**DOI:** 10.3389/finsc.2026.1891174

**Published:** 2026-08-11

**Authors:** Jang-Hyeon Kim, Se-Ji Lee, Ji-Yeon Kim, Mi-Sun Yoo, So Youn Youn, Yun Sang Cho, Hyang-Sim Lee

**Affiliations:** Laboratory of Parasitic and Honeybee Diseases, Bacterial Disease Division, Department of Animal and Plant Health Research, Animal and Plant Quarantine Agency, Gimcheon-si, Gyeongsangbuk-do, Republic of Korea

**Keywords:** active surveillance, *Apis mellifera*, black queen cell virus, co-infection dynamics, deformed wing virus, seasonal surveillance, South Korea, *Varroa destructor*

## Abstract

Honey bee colony losses are an increasing global concern, yet nationwide pathogen-resolved surveillance data integrating molecular diagnostics with field-based mite monitoring remain limited. This study presents the first national active surveillance program for honey bee diseases in South Korea, combining standardized field inspections with RT-qPCR-based pathogen detection. A total of 1,715 samples were collected from 107 apiaries during three seasonal surveillance rounds in 2025: Round 1 (post-overwintering; March–May), Round 2 (post-honey-harvest; July–August), and Round 3 (pre-overwintering; October–November). Both *Apis mellifera* and *Apis cerana* colonies were included in the analysis. Fourteen target honey bee pathogens were screened using RT-qPCR assays (Ct ≤ 35), and *Varroa destructor* infestation status in *A. mellifera* apiaries was evaluated through brood inspection or powdered-sugar roll testing depending on seasonal field conditions. DWV prevalence increased progressively from 70.1% in Round 1 (post-overwintering) to 90.4% in Round 2 (post-honey-harvest) and 97.0% in Round 3 (pre-overwintering), coinciding with increasing *Varroa* detection in *A. mellifera* apiaries (28.1%, 85.4%, and 87.9%, respectively). Israeli acute paralysis virus (IAPV) also increased progressively across the three surveillance rounds (18.3%, 40.1%, and 47.7%). In contrast, black queen cell virus (BQCV), sacbrood virus (SBV), *Vairimorpha* spp. (formerly *Nosema* spp.), and chalkbrood showed declining trends toward autumn. Co-infection patterns shifted seasonally. Although the mean number of detected pathogens per sample decreased from 2.76 to 2.02, co-detection of DWV and IAPV increased markedly from 15.3% to 47.0%. In *A. cerana* apiaries, SBV remained highly prevalent throughout the surveillance rounds. These findings indicate that the late-season expansion of the *Varroa*–DWV–IAPV complex represents a major epidemiological feature of Korean apiculture and coincides with the critical period of winter-bee production. This nationwide surveillance framework provides an evidence-based foundation for seasonal honey bee disease management and optimized timing of *Varroa* control interventions in temperate apicultural systems.

## 1 Introduction

Honey bees (*Apis mellifera* and *Apis cerana*) are indispensable pollinators that underpin global food security and agricultural productivity. The number of registered apiaries in South Korea declined from 26,805 in 2022 to 26,686 in 2023 (1), and unexplained mass-mortality events have been reported with increasing frequency across multiple regions. A thorough understanding of the biological drivers behind these losses is therefore urgently needed to safeguard the beekeeping industry and the ecosystem services provided by honey bees.

Viral infections represent a major threat to honey bee health at all developmental stages, from eggs and larvae to pupae and adults (2). More than 30 viruses have been identified in honey bees (3, 4), of which seven are considered the most prevalent and economically significant in modern apiculture: acute bee paralysis virus (ABPV), black queen cell virus (BQCV), chronic bee paralysis virus (CBPV), deformed wing virus (DWV), Israeli acute paralysis virus (IAPV), Kashmir bee virus (KBV), and sacbrood virus (SBV) (2). Diagnosis based solely on clinical signs is unreliable because infected bees are frequently asymptomatic at low viral loads, different viruses can produce overlapping symptoms, and co-infections with multiple pathogens are common. Therefore, molecular diagnostics, such as PCR, are essential for accurate disease surveillance.

In addition to viral pathogens, fungal and bacterial agents that cause brood diseases pose a substantial threat to colony health. Chalkbrood and stonebrood, caused by *Ascosphaera apis* and *Aspergillus* spp. (including *A. flavus*), respectively, infect and weaken larval brood (5). American foulbrood (AFB) and European foulbrood (EFB), caused by *Paenibacillus larvae* and *Melissococcus plutonius*, respectively, are notifiable diseases and remain among the leading causes of larval mortality and colony loss (6, 7).

Parasitic mites are recognized as the most damaging external parasites of honey bees globally (8). *Varroa destructor* feeds on adult bees and developing brood, suppressing immune function and colony strength, and serves as the primary biological vector for multiple honey bee viruses — most notably DWV (9, 10). *Tropilaelaps mercedesae* (formerly misidentified as *T. clareae* in the East Asian region; 11), which is increasingly prevalent in Asian apiculture, similarly transmits viral pathogens and accelerates colony decline (12). Microsporidian infection caused by *Vairimorpha* spp. (*formerly Nosema* spp.), particularly *Vairimorpha ceranae* (*formerly Nosema ceranae*) (13), is an important disease affecting honey bee colonies, shortening adult lifespan, impairing brood rearing and foraging behavior, and reducing colony productivity.

South Korea hosts both the imported Western honey bee *A. mellifera* and the native Asian honey bee *A. cerana*, each presenting distinct epidemiological profiles. SBV has been consistently identified as the predominant pathogen responsible for mass colony losses in *A. cerana* (14, 15), likely reflecting the species-specific susceptibility of the Asian honey bee to this iflavirus. Despite the dual-species nature of Korean apiculture, most published disease surveys have focused on *A. mellifera*, leaving the epidemiology of *A. cerana* pathogens comparatively under-characterized.

Conventional honey bee disease surveillance in many countries, including the U.S. Bee Informed Partnership (BIP), relies primarily on beekeeper questionnaires and passive reporting, which cannot capture pathogen-level dynamics or seasonal shifts in disease burden at the resolution needed for targeted intervention (16). The pan-European EPILOBEE program (17) likewise combines field inspection with seroprevalence diagnostics but does not include comprehensive multiplex molecular screening. In South Korea, structured active surveillance combining standardized field inspection protocols with laboratory-confirmed molecular diagnostics has not previously been conducted at the national scale.

The 2025 National Honey Bee Disease Active Surveillance Program was therefore designed to address three specific objectives: (i) to characterize the seasonal prevalence of 14 major honey bee pathogens — covering bacterial, fungal, microsporidian, and viral agents — across three critical management periods (post-overwintering, post-honey-harvest, and pre-overwintering); (ii) to characterize seasonal *V. destructor* detection patterns and its temporal association with viral disease burden, particularly DWV; and (iii) to provide evidence-based recommendations for optimal disease-management timing for South Korea’s temperate, seasonally driven beekeeping context.

## 2 Materials and methods

### 2.1 Study design and sampling

In 2025, a nationwide active surveillance program was conducted across eight provincial regions in South Korea. Apiaries were enrolled from the following regions: Gyeonggi (GG), Gangwon (GW), Chungcheongbuk-do (CB), Chungcheongnam-do (CN), Gyeongsangbuk-do (GB), Gyeongsangnam-do (GN), Jeolla (JL; combining Jeollabuk-do, Jeollanam-do, and Gwangju Metropolitan City), and Jeju (JJ). Both Western honey bees (*A. mellifera*) and Asian honey bees (*A. cerana*) were included. Colonies were monitored at three seasonal time points corresponding to critical management transitions in Korean apiculture:

Round 1 (post-overwintering; March–May): 107 apiaries (18 A*. cerana* + 89 *A. mellifera*);
Round 2 (post-honey-harvest; July–August): 107 apiaries (18 A*. cerana* + 89 *A. mellifera*);
Round 3 (pre-overwintering; October–November): 123 apiaries (16 A*. cerana* + 107 *A. mellifera*).

The increase in Round 3 reflects additional apiary enrolment, primarily in Jeolla (JL) and Gangwon (GW) provinces.

Multiple colonies were examined per apiary during each visit. From each sampled colony, ≥10 adult bees and ≥10 larvae (when brood was present) were collected in sterile containers and stored at −80 °C until analysis. *Varroa destructor* infestation was assessed on-site by examining adult worker bees for the presence of mites (as described in Section 2.5). The regional distribution of the surveyed apiaries is summarized in **Table 1**.

**Table 1:** Regional distribution of surveyed apiaries by honey bee species and surveillance round, South Korea, 2025.

Region	Round 1			Round 2			Round 3		
	Cer	Mel	Sub	Cer	Mel	Sub	Cer	Mel	Sub
GG	—	7	7	—	2	2	—	7	7
GW	11	4	15	10	7	17	7	10	17
CB	—	8	8	—	11	11	—	8	8
CN	—	14	14	—	14	14	—	12	12
GB	7	26	33	8	27	35	9	32	41
GN	—	13	13	—	10	10	—	10	10
JL	—	7	7	—	7	7	—	18	18
JJ	—	10	10	—	11	11	—	10	10
**Total**	**18**	**89**	**107**	**18**	**89**	**107**	**16**	**107**	**123**

Cer, *A. cerana*; Mel, *A. mellifera*. An apiary was identified by a unique beekeeper name.Bold values indicate totals.

A total of 1,715 individual samples were processed for laboratory analysis (Round 1: 458; Round 2: 634; Round 3: 623). The breakdown by region, bee species, and sample type is provided in **Table 2**. Abbreviations: Cer-A = *A. cerana* adult; Cer-L = *A. cerana* larva; Mel-A = *A. mellifera* adult; Mel-L = *A. mellifera* larva; Sub = round subtotal.

**Table 2:** Number of honey bee samples submitted for pathogen analysis by region, sample type, and surveillance round, South Korea, 2025.

Region	Round 1					Round 2					Round 3				
	Cer-A	Cer-L	Mel-A	Mel-L	Sub	Cer-A	Cer-L	Mel-A	Mel-L	Sub	Cer-A	Cer-L	Mel-A	Mel-L	Sub
GG	—	—	19	19	38	—	—	6	6	12	—	—	23	12	35
GW	24	14	8	8	54	30	21	21	18	90	23	2	31	14	70
CB	—	—	17	17	34	—	—	33	32	65	—	—	17	4	21
CN	—	—	34	32	66	—	—	42	39	81	—	—	40	19	59
GB	18	2	62	54	136	25	5	82	81	193	26	—	97	71	194
GN	—	—	36	23	59	—	—	42	36	78	—	—	37	35	72
JL	—	—	14	14	28	—	—	22	21	43	—	—	63	38	101
JJ	—	—	23	20	43	—	—	36	36	72	—	—	36	35	71
**Total**	**42**	**16**	**213**	**187**	**458**	**55**	**26**	**284**	**269**	**634**	**49**	**2**	**344**	**228**	**623**

Cer-A, *A. cerana* adult; Cer-L, *A. cerana* larva; Mel-A, *A. mellifera* adult; Mel-L, *A. mellifera* larva; Sub, round subtotal.Bold values indicate totals.

### 2.2 Nucleic acid extraction

Nucleic acids were extracted using a Maxwell^®^ RSC Viral Total Nucleic Acid Purification Kit (Promega, Madison, WI, USA). Two adult bees or five larvae per sample were combined with 600 µL of phosphate-buffered saline in a tissue-homogenizing tube with steel beads (diameter = 2.381 mm; SNC, Hanam, South Korea) and homogenized using a Precellys 24 tissue homogenizer (Bertin Instruments, Montigny-le-Bretonneux, France) for four 15-s cycles at 5,000 rpm. Three hundred microliters of the homogenate, 300 µL of lysis buffer, and 30 µL of proteinase K solution were then combined in a 1.5-mL microcentrifuge tube and incubated at 56 °C for 10 min. Nucleic acids were purified using the automated Maxwell^®^ RSC instrument according to the manufacturer’s instructions. A final volume of 100 µL of total nucleic acid was used as a template for pathogen detection.

### 2.3 Detection of honey bee viral pathogens

The LiliF™ SBV/KSBV/DWV/BQCV reverse-transcription real-time polymerase chain reaction (RT-qPCR) kit and LiliF™ ABPV/KBV/IAPV/CBPV RT-qPCR kit (iNtRON Biotechnology Inc., Seongnam, Korea) were used to detect DWV, SBV, BQCV, ABPV, KBV, IAPV, and CBPV (**Table 3**). The RT-qPCR program consisted of reverse transcription at 45 °C for 30 min, initial denaturation at 95 °C for 10 min, and 40 PCR cycles of 15 s at 95 °C and 1 min at 62 °C. Positive samples were identified by a Ct value ≤ 35. All procedures followed the standardized laboratory protocols of the Animal and Plant Quarantine Agency. The commercial RT-qPCR kits used in this study are validated diagnostic assays for which analytical sensitivity (limit of detection) and target specificity were established by the manufacturer, and the Ct ≤ 35 positivity threshold followed the manufacturer’s validated cut-off. To monitor amplification performance and detect potential contamination, each RT-qPCR run included a no-template (negative) control and a positive control; runs were accepted only when the positive control amplified within its expected range and the no-template control showed no amplification.

**Table 3:** Primer and probe sequences used to detect viral pathogens.

No.	Target	Primer	Sequence (5′ → 3′)	Target gene
1	SBV	SBV-F SBV-R Probe	AGAAGACATTTGATACAGTGGACTC GGAATTCCAGATTCTTCGTCCAC FAM-GATTTGTTTAATGGTTGGGTTTCTGGTA-BHQ-1	Polyprotein gene, 131 bp
2	DWV	DWV-F DWV-R Probe	TTCAACTCGGCTTTCTACGG GTGTCTTTTTCTCTTTCTGACACC ROX-ATGTCAACATTGGTATGCTCCGTTGAC-BHQ-2	Polyprotein gene, 170 bp
3	BQCV	BQCV-F BQCV-R Probe	CCTTTGGCAATAGAACAAATACC GTGGCTATATCGAGATTATTCCG Cy5-AGTCGCAGAGTTCCAAATACCGTACTATG-BHQ-3	Capsid, 143 bp
4	ABPV	ABPV-F ABPV-R Probe	TGCCCTATTTAGGGTGAGGAG GGAGTTTCCACATCATGAAAGG FAM-CTCTGAAGAAAACTCAGTTGAAACGGAAC-BHQ-1	Capsid, 239 bp
5	KBV	KBV-F KBV-R Probe	ACCAGGAAGTATTCCCATGGTAAG TGGAGCTATGGTTCCGTTCAG HEX-CCGCAGATAACTTAGGACCAGATCAATCACA-BHQ-1	Capsid, 79 bp
6	IAPV	IAPV-F IAPV-R Probe	TGCCCTATTTAGGGTGAGGAG GGAGTTTCCACATCATGAAAGG ROX-ACTAGTGAGAACTCGGTTGAGACCCAAG-BHQ-2	Capsid, 245 bp
7	CBPV	CBPV-F CBPV-R Probe	CGCAAGTACGCCTTGATAAAGAAC ACTACTAGAAACTCGTCGCTTCG Cy5-TCAAGAACGAGACCACCGCCAGTTC-BHQ-3	RdRp, 101 bp

ABPV, acute bee paralysis virus; BQCV, black queen cell virus; CBPV, chronic bee paralysis virus; DWV, deformed wing virus; IAPV, Israeli acute paralysis virus; KBV, Kashmir bee virus; SBV, sacbrood virus.

### 2.4 Detection of fungal, bacterial, microsporidian, and parasitic pathogens

The POBGEN™ Bee Pathogen Detection Kits (DB-A2 and DB-B2; POSTBIO Inc., Guri, Korea) were used to detect fungal pathogens (*Aspergillus flavus*, the causative agent of stonebrood, and *Ascosphaera apis*, the causative agent of chalkbrood), microsporidians (*Vairimorpha ceranae*), bacterial pathogens (*Paenibacillus larvae*, AFB; and *Melissococcus plutonius*, EFB), and parasitic agents (the tracheal mite *Acarapis woodi* and the phorid fly *Apocephalus borealis*) (**Table 4**). PCR was performed at 95 °C for 5 min for initial denaturation, followed by 40 cycles of 10 s at 95 °C and 30 s at 60 °C. Positive samples were defined as those with Ct values ≤ 35. All procedures followed the standardized laboratory protocols of the Animal and Plant Quarantine Agency. As for the viral assays, these commercial detection kits are manufacturer-validated for sensitivity and specificity, and every PCR run incorporated a no-template (negative) control and a positive control to verify assay performance and exclude cross-contamination.

**Table 4:** Primers and probe sequences used to detect brood diseases and parasites.

No.	Target	Primer	Sequence (5′ → 3′)	Target gene
1	*Paenibacillus larvae* (AFB)	AFB-F AFB-R Probe	AAATCATCATGCCCCTTATG CGATTACTAGCAATTCCGACT FAM-CGTACTACAATGGCCGGTACAACG-BHQ-1	16S rRNA, 158 bp
2	*Melissococcus plutonius* (EFB)	EFB-F EFB-R Probe	TGTTGTTAGAGAAGAATAGGGGAA CGTGGCTTTCTGGTTAGA Cy5-AGAGTAACTGTTTTCCTCGTGACGGT-BHQ-3	16S rRNA, 69 bp
3	*Apocephalus borealis* (phorid fly)	Phorid-F Phorid-R Probe	CCTCTGTTCTACTTTCATTGGTTTAT GAGRGCCATAAAAGTAGCTACACC JOE-GGCATTAGTATTACGACGCGAGAGGTG-BHQ-1	18S rRNA, 255 bp
4	*Acarapis woodi* mite	ACAR-F ACAR-R Probe	CAGTAGGGCTAGATATCGATACCCGAGCTT TGAGCTACAACATAATATCTGTCATGAAGA TexasRed-CAATCCACCTACAGAAAATAAAAATAAAAATCC-BHQ2	COI, 247 bp
5	*Vairimorpha ceranae* (formerly *Nosema ceranae*)	*Nosema*-F *Nosema*-R Probe	CGGATAAAAGAGTCCGTTACC GAGCAGGGTTCTAGGGAT FAM-CGTTACCCTTCGGGGAATCTTC-BHQ-1	LSU rRNA, 249 bp
6	*Aspergillus flavus* (stonebrood)	ASP-F ASP-R	GCTGCCCATCAAGCACGG CCTACAGAGCGGGTGACAAAG JOE-TGTGTGTTGGGTCGTCGTCCCCTCTC-BHQ-1	ITS2, 127 bp
7	*Ascosphaera apis* (chalkbrood)	ASCO-F ASCO-R Probe	ATTGCGCCCTCTGGTATTC CCACTAGAAGTAAATGATGGTTAGA TexasRed-GCTTGAGGGTTGCAATGACGCTCG BHQ-2	ITS2, 215 bp

AFB, American foulbrood; EFB, European foulbrood.

### 2.5 *Varroa destructor* infestation

During Round 1 (post-overwintering), mite populations were generally low, and colonies were rapidly expanding for spring honey production. Because beekeepers were reluctant to sample approximately 300 adult worker bees for powdered-sugar roll testing during this sensitive colony-growth period, *Varroa* screening was instead performed by visual examination of ≥10 capped brood cells, including drone brood when available. Using this approach, *Varroa* mites were detected in 25 of the 89 A*. mellifera* apiaries (28.1%). During Round 2 (post-honey-harvest) and Round 3 (pre-overwintering), when mite populations increased substantially, *Varroa* infestation status was assessed using powdered-sugar roll tests on approximately 300 adult bees from three colonies per apiary.

### 2.6 Statistical analysis

Sample-level pathogen prevalence was calculated as the proportion of positive samples among all samples within each surveillance round and honey bee species. Apiary-level (farm-level) prevalence was defined as the proportion of apiaries with at least one positive sample. An apiary was identified by a unique beekeeper name. Co-infection load per sample was quantified as the number of distinct pathogens detected at the sample level; SBV was treated as a single virus. Pairwise pathogen co-occurrence was summarized as the proportion of samples positive for both members of each pair within each round. The association between round-level V. *destructor* and DWV prevalence across rounds was first summarized descriptively. To formally test the *Varroa*–DWV association at the sample level while accounting for non-independence of samples collected within the same apiary, a generalized linear mixed model (GLMM) with a binomial error structure and logit link was fitted, with DWV status (positive/negative) as the response, *V. destructor* detection (presence/absence) and surveillance round as fixed effects, and apiary (beekeeper identity) as a random intercept. Because the Round 1 mite assessment used capped-brood inspection whereas Rounds 2–3 used powdered-sugar rolls, *V. destructor* was modeled as a binary presence/absence term rather than as a quantitative count to reduce method-dependent bias. Model estimates are reported as odds ratios (ORs) with 95% confidence intervals; a complementary cluster-robust (GEE) logistic model gave consistent results. Mean co-infection counts between rounds were compared using the Kruskal–Wallis rank-sum test, followed by pairwise Wilcoxon rank-sum tests with Bonferroni correction. Descriptive prevalence and co-infection summaries were computed in Python 3 using pandas. The GLMM, GEE, and Kruskal–Wallis analyses were performed in R version 4.6.1 (18) using the lme4 (v2.0.1) and geepack (v1.3.13) packages. Two-sided p-values < 0.05 were considered statistically significant.

## 3 Results

### 3.1 Seasonal changes in pathogen detection rates

**Figure 1:**
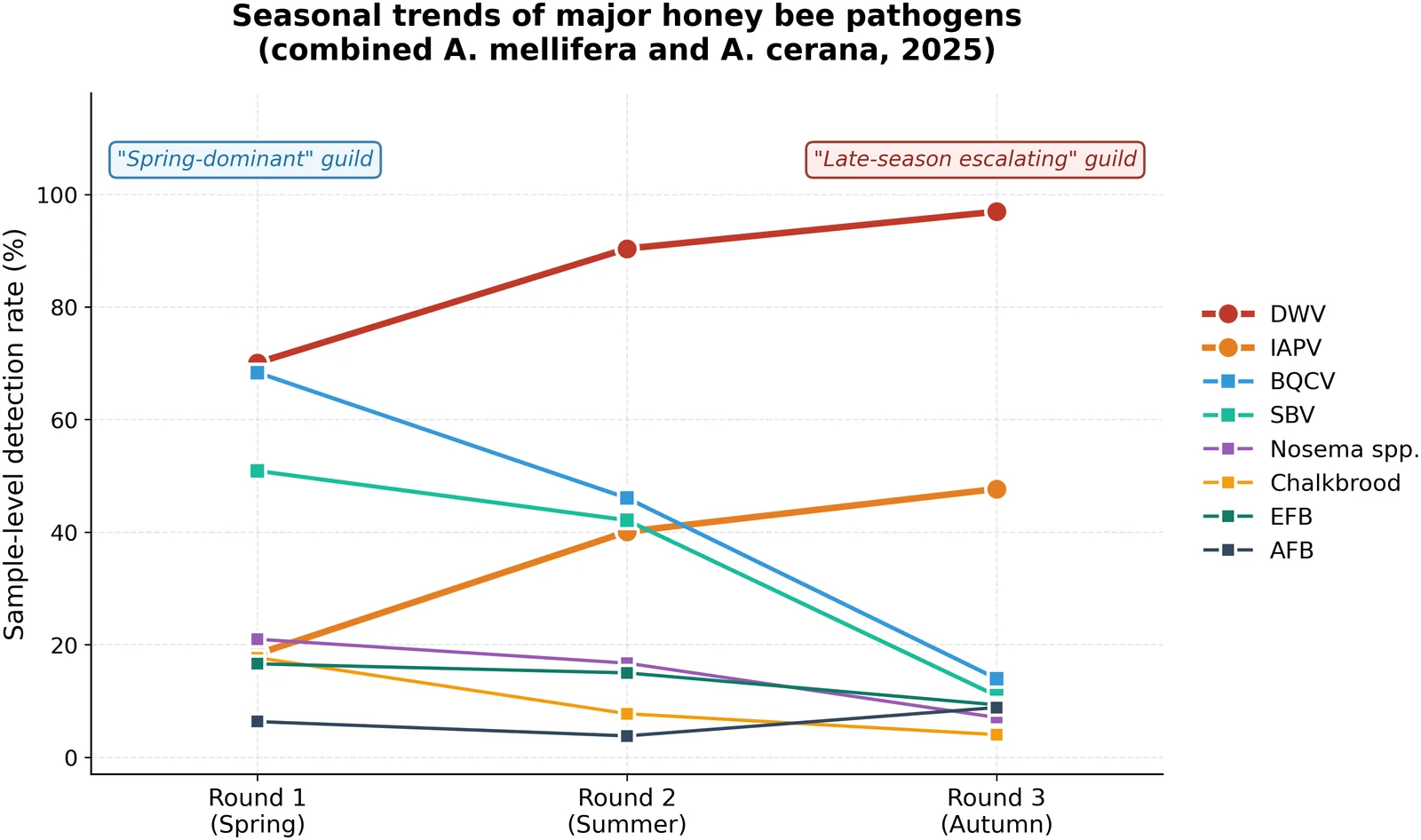
Seasonal trends of major honey bee pathogens across three surveillance rounds (sample-level detection rate, %, combined *A. mellifera* and *A. cerana*, 2025). DWV and IAPV (“late-season escalating guild”) rose progressively from spring to autumn; BQCV, SBV, *Vairimorpha* spp., chalkbrood, EFB, and AFB (“spring-dominant guild”) declined or remained stable. Detection threshold: Ct ≤ 35.

Across 1,715 samples collected over three surveillance rounds, distinct seasonal trends were identified for each pathogen category (**Table 5**, **Figure 1**). Viral pathogens showed divergent dynamics: DWV and IAPV increased progressively across rounds, whereas BQCV, SBV, and ABPV declined toward autumn.

**Table 5:** Seasonal sample-level detection rates (%) of major honey bee pathogens across three surveillance rounds, South Korea, 2025.

Pathogen	Abbrev.	Category	Round 1	Round 2	Round 3
Deformed wing virus	DWV	Virus	70.1	90.4	97.0
Israeli acute paralysis virus	IAPV	Virus	18.3	40.1	47.7
Black queen cell virus	BQCV	Virus	68.3	46.1	14.0
Sacbrood virus	SBV	Virus	50.9	42.1	10.9
Chronic bee paralysis virus	CBPV	Virus	4.6	1.9	2.1
Acute bee paralysis virus	ABPV	Virus	1.1	0.2	0.0
Kashmir bee virus	KBV	Virus	0.4	0.0	0.0
*Vairimorpha ceranae*	*V. ceranae*	Microsporidian	21.0	16.7	7.1
*Ascosphaera apis* (chalkbrood)	ASCO	Fungal	17.7	7.7	4.0
*Aspergillus flavus* (stonebrood)	ASP	Fungal	0.4	0.5	0.8
*Melissococcus plutonius* (EFB)	EFB	Bacterial	16.6	15.0	9.3
*Paenibacillus larvae* (AFB)	AFB	Bacterial	6.3	3.8	8.8

Data represent combined *A. mellifera* and *A. cerana* samples.

Deformed wing virus (DWV) was the most prevalent pathogen in all three rounds. Detection rates rose from 70.1% in Round 1 to 90.4% in Round 2 and reached 97.0% in Round 3, approaching universal prevalence in the pre-overwintering period. IAPV showed a parallel increasing trend, rising from 18.3% in Round 1 to 40.1% in Round 2 and 47.7% in Round 3.

BQCV was the second most prevalent pathogen in spring (68.3%) but declined sharply to 46.1% in Round 2 and 14.0% in Round 3. SBV followed a similar trajectory (50.9% → 42.1% → 10.9%), as did ABPV (1.1% → 0.2% → 0.0%) and chalkbrood (ASCO; 17.7% → 7.7% → 4.0%). *Vairimorpha* spp. also decreased consistently (21.0% → 16.7% → 7.1%), suggesting higher microsporidian burden in the post-overwintering period.

Bacterial brood diseases remained at comparatively low levels throughout the year. EFB prevalence decreased from 16.6% (Round 1) to 9.3% (Round 3), while AFB prevalence declined from 6.3% to 3.8% in Round 2, before increasing slightly to 8.8% in Round 3. Stonebrood (ASP) remained below 1% across all rounds (**Table 5**). In contrast, two target pathogens—*Apocephalus borealis* (phorid fly) and *Acarapis woodi*—were not detected during the surveillance period and were therefore excluded from **Table 5**. KBV was detected in only two samples in Round 1 (0.4%) and was not detected thereafter (**Table 5**). Their absence is consistent with previous nationwide surveillance studies in South Korea that reported either non-detection or extremely low prevalence of these pathogens and parasites.

### 3.2 Seasonal dynamics of *Varroa destructor* infestation

*Varroa destructor* detection in *A. mellifera* apiaries increased markedly across the three surveillance rounds (**Table 6**). The apiary-level detection rate rose from 28.1% in spring to 85.4% in Round 2 and 87.9% in autumn, indicating the rapid expansion of *Varroa* infestation during the active beekeeping season. It should be noted, however, that the Round 1 estimate was obtained by visual inspection of capped brood, whereas Rounds 2 and 3 used powdered-sugar rolls on adult bees (Section 2.5). Because these methods differ in their biological target and sensitivity, the round-to-round values should be read as a qualitative seasonal increase in *Varroa* detection rather than as a strictly standardized quantitative time series.

**Table 6:** *Varroa destructor* infestation in *A. mellifera* apiaries across three surveillance rounds, South Korea, 2025.

Round	Total apiaries	*A. mellifera* apiaries	Positive apiaries	Detection rate (%)
Round 1	107	89	25	28.1
Round 2	107	89	76	85.4
Round 3	123	107	94	87.9

### 3.3 Association between *Varroa* infestation and DWV prevalence

A consistent temporal association was observed between *Varroa* apiary-level detection rate and DWV sample-level prevalence across the three rounds. As *Varroa* infestation increased from 28.1% to 85.4% to 87.9%, DWV prevalence rose in parallel from 70.1% to 90.4% to 97.0%. The relationship is consistent with the established role of *V. destructor* as the principal biological vector amplifying DWV within Korean honey bee colonies (10). Beyond these detection-rate trends, the underlying viral load also increased markedly: among DWV-positive *A. mellifera* samples, the median DWV Ct value fell from 28.1 (Round 1) to 23.2 (Round 2) to 18.0 (Round 3) (**Table 7**), indicating that DWV was not merely detected more frequently but was present at progressively higher titer as the season advanced. By contrast, the median Ct values of IAPV, BQCV, and SBV remained comparatively stable across rounds (**Table 7**), underscoring the specificity of the late-season DWV amplification. Because the round-level detection-rate association is based on only three aggregate time points, it is presented descriptively rather than as inferential evidence; the sample-level Ct trends provide a more robust, quantitative line of support for the *Varroa*–DWV relationship. Only *A. mellifera* samples were included in this analysis because *Varroa* surveillance was conducted exclusively in *A. mellifera* apiaries. To test this association at the sample level while accounting for clustering of samples within apiaries, a binomial GLMM was fitted with apiary as a random intercept (1,525 *A. mellifera* samples from 150 apiaries). After adjusting for surveillance round, *V. destructor* detection was independently associated with significantly higher odds of DWV positivity (odds ratio [OR] = 1.97, 95% CI 1.15–3.37, p = 0.013; **Table 8**). Surveillance round was also strongly associated with DWV positivity (Round 3 vs Round 1: OR = 12.92, 95% CI 6.84–24.43, p < 0.001), consistent with the marked late-season increase in viral load described above. A complementary cluster-robust GEE logistic model produced closely consistent estimates (*V. destructor*: OR = 1.90, 95% CI 1.14–3.17, p = 0.014; **Table 8**), indicating that the Varroa–DWV association is robust to the modeling approach and is not an artifact of apiary-level clustering or of the three-point round-level summary.

**Table 7:** Seasonal median Ct values of major viral pathogens in virus-positive *A. mellifera* samples.

Virus	Round 1 median Ct (IQR), n	Round 2 median Ct (IQR), n	Round 3 median Ct (IQR), n
DWV	28.1 (23.8–31.4), 302	23.2 (18.2–27.1), 503	18.0 (12.4–23.1), 558
IAPV	29.0 (24.8–32.3), 83	30.9 (26.4–33.1), 237	29.2 (24.8–32.6), 293
BQCV	27.0 (22.4–30.3), 277	27.9 (25.8–30.5), 240	28.0 (23.6–31.0), 78
SBV	31.8 (28.6–33.3), 175	30.6 (28.0–32.4), 210	30.8 (27.0–33.1), 34

Lower Ct values indicate higher relative viral loads. Median Ct values were calculated from RT-qPCR-positive samples for each respective virus (Ct ≤ 35). Values are presented for each surveillance round as median (IQR); n, number of positive samples.

**Table 8:** Association between *V. destructor* detection and DWV positivity in *A. mellifera* samples, estimated from a binomial generalized linear mixed model (GLMM) and a complementary cluster-robust generalized estimating equation (GEE) model.

Fixed effect	GLMM OR	95% CI	p	GEE OR	95% CI	p
*V. destructor* detected (vs not detected)	1.97	1.15–3.37	0.013	1.90	1.14–3.17	0.014
Round 2 (vs Round 1)	2.96	1.92–4.56	<0.001	2.69	1.67–4.33	<0.001
Round 3 (vs Round 1)	12.92	6.84– 24.43	<0.001	10.94	5.20– 23.00	<0.001

The GEE used an exchangeable working correlation structure with apiary as the clustering variable. Odds ratios (ORs) and 95% confidence intervals (CIs) are presented. Reference categories were *V. destructor* not detected, and Round 1.

### 3.4 Co-infection patterns and seasonal changes in pathogen complexity

**Figure 2:**
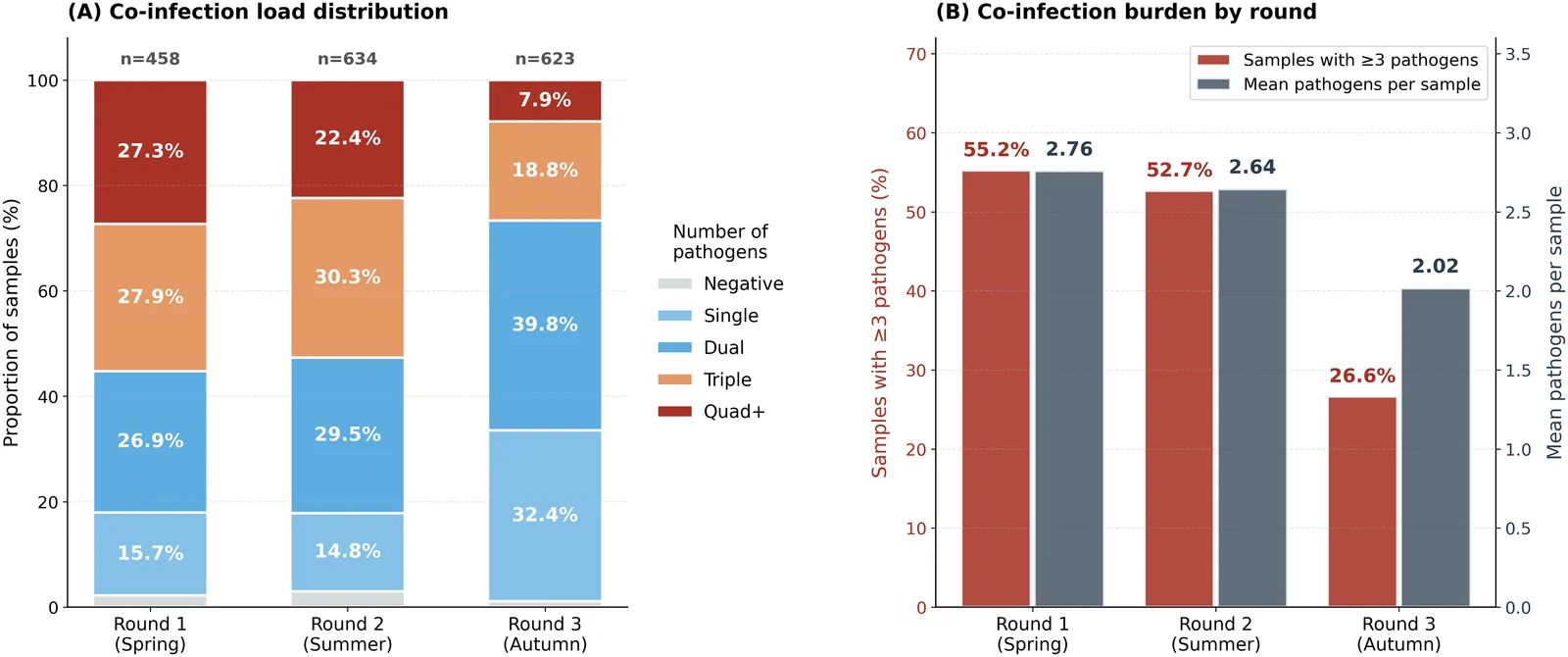
Co-infection load distribution across surveillance rounds. **(A)** Stacked bar showing the proportion of samples carrying 0 (Negative), 1 (Single), 2 (Dual), 3 (Triple), or ≥4 (Quad+) pathogens. **(B)** Samples with ≥3 pathogens (left axis) and mean number of pathogens per sample (right axis). Both metrics decline from Round 1 to Round 3 — a reflection of the dropout of the spring-dominant pathogen guild, not of improving colony health.

Co-infection burden displayed a counter-intuitive pattern. Negative samples (zero pathogens detected) remained a small minority throughout — 2.2% in Round 1 (post-overwintering), 3.0% in Round 2 (post-honey-harvest), and 1.1% in Round 3 (pre-overwintering) — confirming that single-agent infections are the exception rather than the rule in Korean honey bee colonies under field conditions. However, the proportion of samples carrying three or more pathogens decreased from 55.2% in Round 1 (post-overwintering) to 52.7% in Round 2 (post-honey-harvest) and 26.6% in Round 3 (pre-overwintering), while the mean number of pathogens per sample declined from 2.76 in spring to 2.64 in Round 2 and 2.02 in Round 3 (Kruskal–Wallis χ² = 126.5, df = 2, p < 0.001). Pairwise comparisons (Wilcoxon rank-sum tests with Bonferroni correction) indicated that Round 1 and Round 2 did not differ significantly (p = 0.70), whereas Round 3 differed from both Round 1 and Round 2 (both p < 0.001), indicating that the reduction in co-infection load occurred specifically in the pre-overwintering period rather than progressively across the season. The full distribution of co-infection load across rounds is shown in **Figure 2**.

**Figure 3:**
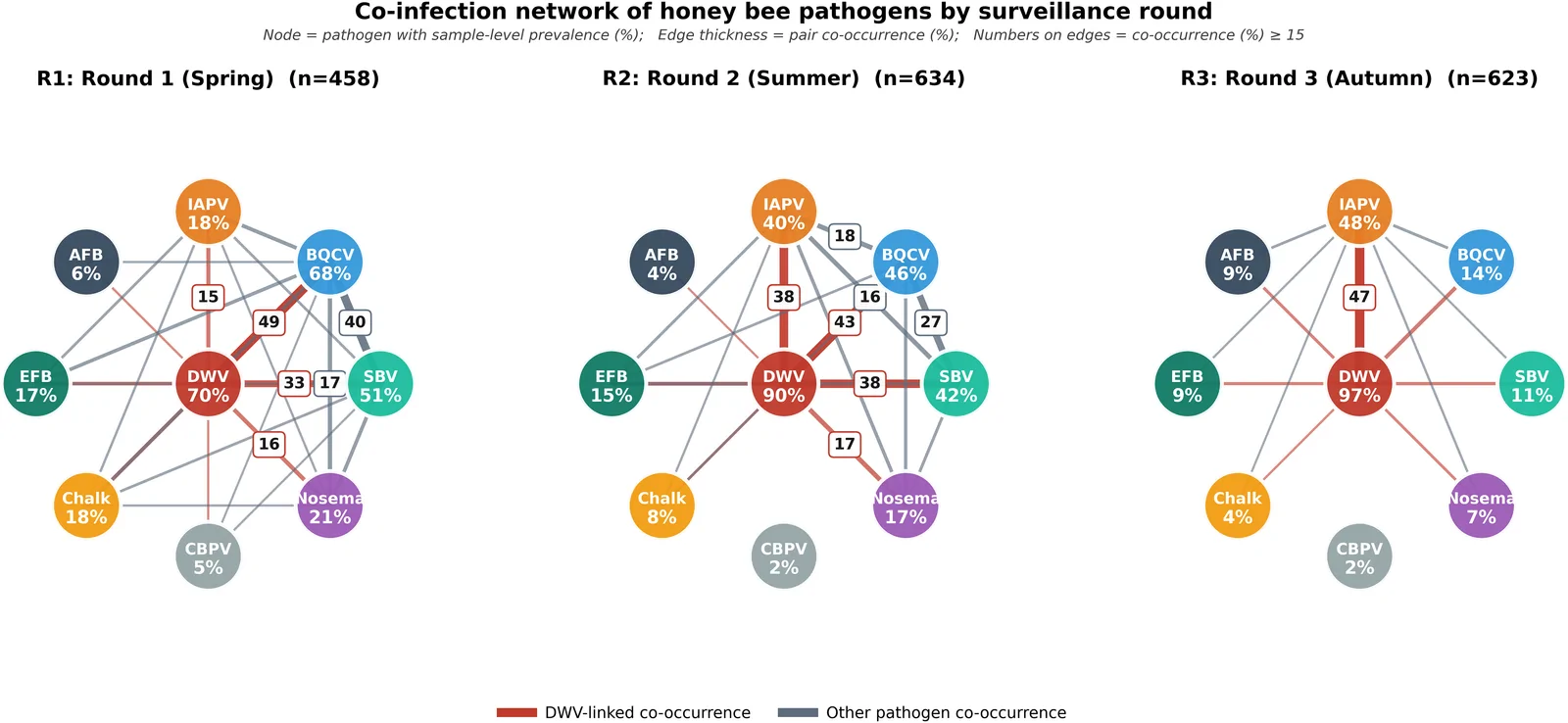
Co-infection network of honey bee pathogens by surveillance round. Each node represents a pathogen, with sample-level prevalence (%) given inside the node and node positions held fixed across the three rounds for direct visual comparison. Edge thickness is proportional to pairwise co-occurrence (% of samples positive for both pathogens), and numerical labels are placed on edges with co-occurrence ≥ 15%. Red edges denote DWV-linked co-occurrence; dark-gray edges denote other pathogen pairs. The spring (R1) network is dominated by BQCV–SBV–DWV interactions; by autumn (R3), only the DWV–IAPV connection remains strongly weighted.

This simplification of the pathogen burden reflects compositional reshuffling rather than an improvement in colony health. The spring-dominant guild (BQCV, SBV, *Vairimorpha*, chalkbrood, and EFB) — which together accounted for the bulk of co-infection complexity in Round 1 (post-overwintering) — declined sharply from Round 1 to Round 3 (**Figure 1**). At the same time, the late-season escalating guild — DWV and IAPV — converged on an ever-larger fraction of colonies. The proportion of samples co-detecting DWV and IAPV more than tripled across rounds (15.3% → 38.3% → 47.0%), while DWV co-detection with BQCV decreased (49.1% → 42.7% → 13.6%) and with SBV decreased (33.0% → 37.5% → 10.1%) (**Figure 3**). Thus, although the average number of pathogens per sample decreased, the residual co-infection profile consolidated around the *Varroa*-mediated DWV–IAPV axis, a numerically simpler but biologically more dangerous configuration for colonies preparing to overwinter.

### 3.5 Disease patterns in Asian honey bees (*Apis cerana*)

**Figure 4:**
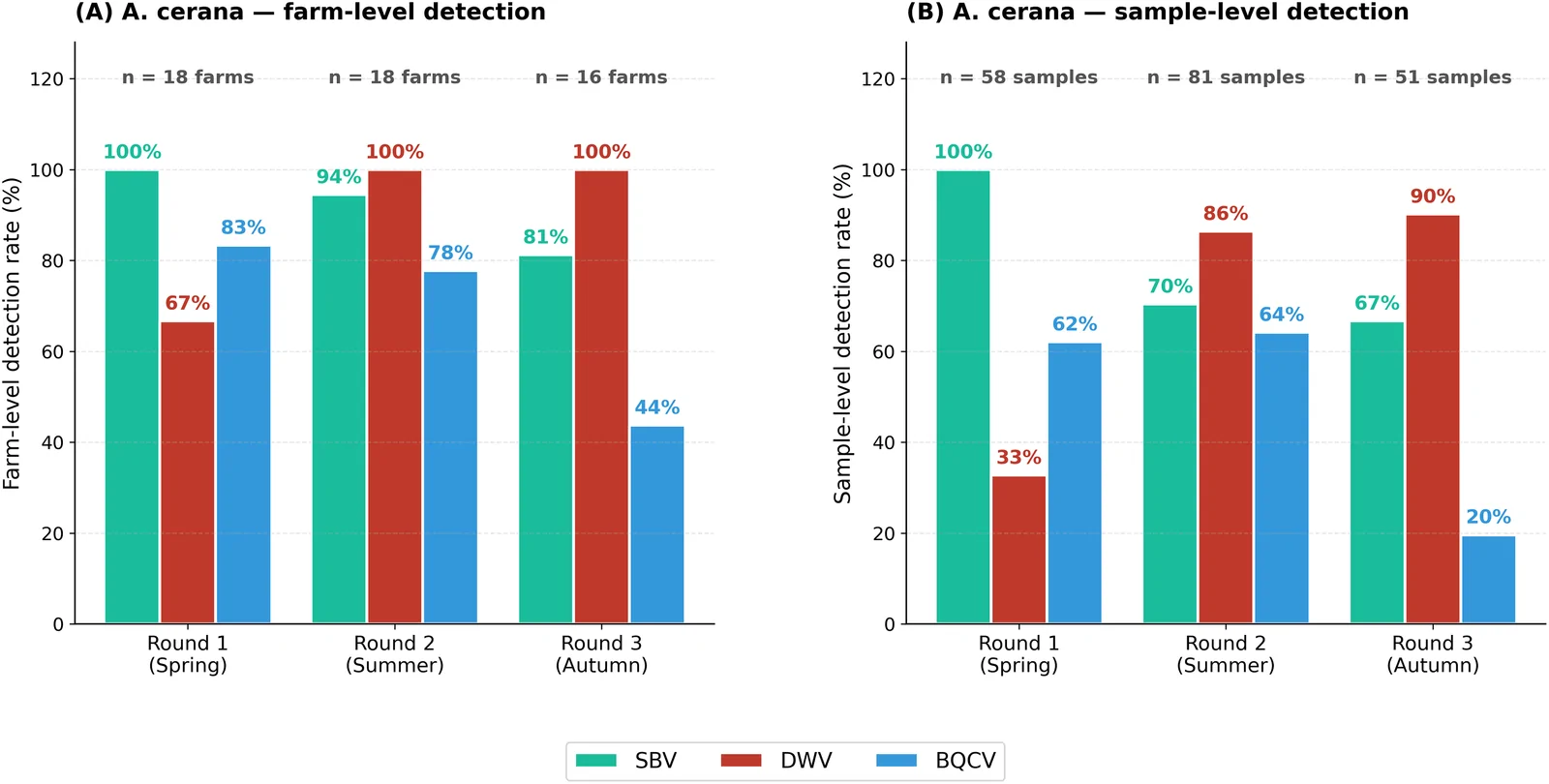
Seasonal dynamics of SBV, DWV, and BQCV in Asian honey bee (*Apis cerana*) apiaries across three surveillance rounds, South Korea, 2025. **(A)** Farm-level detection rates. **(B)** Sample-level detection rates. SBV maintained near-universal farm-level prevalence in all rounds; DWV escalated from Round 1 to Round 3 in parallel with the pattern observed in *A. mellifera*; BQCV declined steeply.

Seasonal viral dynamics in *A. cerana* apiaries differed substantially from those in *A. mellifera*. The most prominent feature was the persistently high prevalence of sacbrood virus (SBV) throughout the surveillance period, with farm-level detection rates of 81.2–100% across all three rounds (**Table 9**, **Figure 4**). Unlike *A. mellifera*, in which SBV declined steeply toward autumn, *A. cerana* colonies maintained near-universal SBV infection regardless of the season, confirming the well-documented species-specific susceptibility of the native Asian honey bee to this pathogen. The prevalence of DWV in *A. cerana* apiaries increased markedly from Round 2 onward. Farm-level rates rose from 66.7% (Round 1; post-overwintering) to 100% (Round 2; post-honey-harvest) and remained at 100% (Round 3; pre-overwintering), while sample-level rates increased from 32.8% to 86.4% to 90.2%. BQCV, by contrast, followed a declining trajectory (farm-level: 83.3% → 77.8% → 43.8%; sample-level: 62.1% → 64.2% → 19.6%), paralleling its pattern in Western honey bee colonies. These opposing trends—an increase in DWV and a decline in BQCV—suggest divergent ecological drivers for the two viruses and indicate that the late-season DWV escalation is a pan-species phenomenon in Korean apiculture, affecting both managed bee species.

**Table 9:** Seasonal farm-level and sample-level prevalence (%) of three major viral pathogens in Asian honey bee (*A. cerana*) apiaries, South Korea, 2025.

Virus	Detection level	Round 1	Round 2	Round 3
SBV	Farm-level (%)	100.0	94.4	81.2
	Sample-level (%)	100.0	70.4	66.7
DWV	Farm-level (%)	66.7	100.0	100.0
	Sample-level (%)	32.8	86.4	90.2
BQCV	Farm-level (%)	83.3	77.8	43.8
	Sample-level (%)	62.1	64.2	19.6

## 4 Discussion

This nationwide active surveillance provides descriptive evidence that the late-season increase of the *Varroa*–DWV–IAPV complex is the dominant seasonal epidemiological pattern associated with colony-health deterioration in South Korean apiculture. Three findings stand out: (i) the existence of two ecologically distinct pathogen guilds with opposite seasonal trajectories; (ii) a counter-intuitive reversal of co-infection complexity from Round 1 (spring) to Round 3 (autumn), which masks the intensification of the highest-risk co-detections; and (iii) the convergence of all three risk axes —*Varroa* infestation, DWV, and IAPV — during the period of winter-bee production.

### 4.1 Two seasonally distinct pathogen guilds

Honey bee pathogens in Korean apiaries followed two contrasting seasonal trajectories (**Figure 1**). A spring-dominant guild (BQCV, SBV, *Vairimorpha* spp., chalkbrood, EFB) peaked in Round 1 and declined sharply by Round 3 — a pattern consistent with brood-borne pathogens whose transmission depends on the rapid turnover of spring brood and increased social interaction in early-season colonies, and which is dampened by the higher temperatures and improved hive ventilation in summer (2, 19). In contrast, a late-season escalating guild dominated by DWV and IAPV rose progressively from Round 1 through Round 3, mirroring the *V. destructor* infestation curve. The temporal coupling of these two guilds, one driven by intrinsic colony dynamics and the other by an external vector, was the central epidemiological feature of Korean honey bee disease in 2025.

### 4.2 Counter-intuitive reversal of co-infection complexity

A counter-intuitive but biologically meaningful pattern emerged from the co-infection data. The simple count of co-detected pathogens per sample decreased from a mean of 2.76 in Round 1 to 2.02 in Round 3, and the proportion of samples carrying three or more pathogens fell from 55.2% to 26.6%. However, this was not evidence of an improvement in colony health. Rather, it reflects the dropout of the spring-dominant guild, leaving the late-season DWV–IAPV pair as the dominant co-infection axis. The proportion of samples co-detecting DWV and IAPV more than tripled across rounds (15.3% → 38.3% → 47.0%), even as overall pathogen diversity contracted (**Figure 3**; full pairwise co-occurrence matrices for all three rounds are provided in **Supplementary Figure 1**). In effect, the complexity of the pathogen burden decreased while its risk concentration intensified — colonies entering winter face a numerically simpler but biologically more dangerous co-infection profile, dominated by two viruses whose amplification depends on a single vector that is simultaneously approaching peak infestation. This finding revises the conventional interpretation of declining co-infection counts as a marker of recovery and underscores the importance of tracking pathogen composition, not just diversity, across seasons.

### 4.3 Mechanism: *Varroa* as biological vector

The mechanistic basis for the late-season virus surge is well established. *Varroa destructor* serves as a competent biological vector for DWV in a non-propagative manner, transmitting viral particles directly into the hemocoel of developing pupae during feeding (10). Vector-mediated transmission selects for virulent DWV variants (20, 21), and the global spread of these virulent strains has been attributed to the *Varroa* invasion itself (22). Our finding that DWV reaches 97% sample-level prevalence by Round 3 in Korean *A. mellifera* — and that 47% of all samples simultaneously carry IAPV — is consistent with a mature, *Varroa*-driven viral landscape rather than with the early stages of viral expansion. This association between *V. destructor* and DWV positivity was confirmed at the sample level using a binomial GLMM that accounted for apiary-level clustering (OR = 1.97; **Table 8**), providing robust support for this mechanism in Korean apiaries.

### 4.4 Winter-bee implications and operational recommendations

The convergence of peak *V. destructor* infestation, near-universal DWV prevalence, and rising IAPV detection in Round 3 coincides precisely with the period of winter-bee production in temperate Korean apiculture (late September through early November). Winter bees (diutinus workers) are physiologically distinct long-lived honey bees that must survive for 4–6 months on stored fat-body reserves and elevated vitellogenin titers (23, 24). Both *V. destructor* parasitism during pupal development and elevated DWV loads have been experimentally shown to shorten worker lifespan and impair immune function in winter bees (25, 26). The pattern observed in this study — a late-season *Varroa* surge coupled to a tightening DWV–IAPV virus complex in the same colonies that must produce winter bees — is the field-level signature of the mechanism proposed by Dainat et al. (25) for honey bee overwintering failure.

From a management perspective, the most critical insight is the mismatch between current beekeeping practices and the observed pathogen-mite dynamics. The dataset reveals that high apiary-level *Varroa* detection does not first emerge in autumn but is already established by the post-honey-harvest summer round, when the majority of *A. mellifera* apiaries are already *Varroa*-positive. Acaricide treatment delivered only after the autumn honey harvest is therefore too late to prevent the cascade described above. The data support a clear operational recommendation: *Varroa* control should begin in late spring and be intensified between late June and early August, with the recognition that the efficacy of synthetic acaricides, such as amitraz, can vary with temperature and brood conditions, and that organic-acid (oxalic, formic) trickling treatments offer a complementary tool during the autumn broodless period. The effectiveness of late-season *Varroa* control may be further compromised by acaricide resistance in Korean *Varroa* populations; resistance-associated mutations and recent national control-policy changes are examined in detail in a companion surveillance study (under review). Continued reliance on single-mode synthetic acaricides may accelerate treatment failure under high mite-pressure conditions, underscoring the value of integrating seasonal infestation monitoring with resistance assessment for sustainable *Varroa* management in Korean apiculture.

### 4.5 *Apis cerana*: persistent endemic SBV burden

The pathogen profiles of *A. cerana* apiaries differed markedly from those of *A. mellifera*. SBV was detected at a near-universal farm-level prevalence (81.2–100%) across all three rounds, with limited seasonal decline. This persistent endemic infection is consistent with the well-documented species-specific susceptibility of the Asian honey bee to this iflavirus, which has caused recurrent nationwide colony collapse in Korean *A. cerana* populations and is associated with reduced hygienic-behavior efficiency relative to *A. mellifera* (14, 15). DWV prevalence in *A. cerana* paralleled the seasonal increase observed in *A. mellifera* (farm-level 66.7% → 100% → 100%), confirming that *Varroa*-mediated DWV amplification is a pan-species phenomenon in Korean apiculture. However, the limited sample size of *A. cerana* (16–18 apiaries per round, with some sample categories represented by very few specimens — for example, only two larval samples in Round 3) precludes formal statistical comparison, and the corresponding *A. cerana* findings should therefore be regarded as preliminary, descriptive observations rather than robust species-level conclusions; continued surveillance with expanded *A. cerana* sampling is warranted, especially given the species’ high conservation value and persistent SBV burden. The parallel late-season rise of DWV in *A. cerana* observed here is consistent with regional evidence that DWV and SBV are shared between *A. mellifera* and *A. cerana* across East and Southeast Asia, where interspecific transmission among co-occurring Apis species has been documented (27, 28). This cross-species circulation underscores the epidemiological relevance of including the native Asian honey bee in national surveillance.

### 4.6 Methodological comparison and study limitations

Methodologically, this study demonstrates the value of an integrated surveillance system that combines standardized field measurements with molecular diagnostics. Compared with the pan-European EPILOBEE program (17), which combines field inspection with seroprevalence diagnostics, the present design adds comprehensive PCR-based pathogen panels alongside on-site *Varroa* inspection, enabling pathogen-specific, quantitative risk assessment at the national scale. Several limitations should be acknowledged. First, the surveillance rounds are snapshots rather than longitudinal follow-ups of individual colonies. Second, although PCR detection is highly sensitive, it does not distinguish between productive (replicating) and latent infections, particularly for DWV (29). Third, the small sample size of *A. cerana* limited species-specific inferences. Fourth, *Varroa* inspection data were not uniformly available across all apiaries; apiaries without recorded inspection results were treated as negative for the apiary-level *Varroa* rate calculations. Fifth, although the sample-level *Varroa*–DWV association was confirmed using a binomial GLMM with apiary as a random intercept (and corroborated by a GEE model), the present design remains cross-sectional; the mite presence/absence predictor does not capture infestation intensity, and causal and dose–response inferences will require longitudinally tracked, individual-colony data with quantitative mite counts. Sixth, the commercial RT-qPCR assays used here detect total DWV but do not discriminate between the DWV-A and DWV-B master variants, which differ in virulence and transmission dynamics; master-variant–specific genotyping was therefore beyond the scope of the present surveillance and represents an important direction for future molecular characterization. Regional molecular surveys nonetheless indicate that DWV-A remains the predominant master variant across East Asia, including China, Japan, and South Korea (27), and that both DWV-A and DWV-B circulate and are shared among co-occurring Apis species in Southeast Asia (28), providing a useful genotypic context for interpreting the total-DWV detection rates reported here. Finally, while the *Varroa*–DWV relationship was modeled formally, the pathogen–pathogen co-occurrence analyses remain descriptive; full multivariable modeling of interactions among all pathogen pairs (e.g., multinomial or network-based mixed models) will benefit from the individual-colony, longitudinally tracked data that subsequent surveillance rounds are designed to generate. In addition, the surveillance captured pathogen and *Varroa* detection but did not record concurrent colony-health indicators (e.g., colony strength, brood viability, or overwintering survival outcomes), so the present data document pathogen circulation rather than its direct consequences for colony health; linking pathogen profiles to quantitative health and survival metrics is an important objective for subsequent rounds.

The 2025 nationwide active surveillance established that honey bee colony health in South Korea is primarily threatened by the late-season escalation of the *Varroa*–DWV–IAPV complex. The synchronized increase in mite infestation and viral prevalence represents a key biological mechanism driving overwintering failure. The nationwide three-round surveillance framework effectively captured seasonal disease dynamics and identified a critical intervention window — substantially earlier than the conventional post-harvest standard practice.

Based on these results, we propose four evidence-based recommendations. First, *Varroa* control should be implemented from late spring onward and intensified between late June and early August in temperate Korean apiculture; complementary organic-acid (oxalic, formic) treatments and brood-interruption strategies should be available as part of an integrated mite management toolkit. Second, nationwide monitoring of acaricide resistance should be institutionalized within the active surveillance program to maintain treatment efficacy over time; resistance-associated mutations and corresponding policy responses are addressed in detail in a companion study (under review). Third, standardized field inspection protocols and PCR-based diagnostics should be retained and expanded within the routine surveillance system. Fourth, beekeeper management variables (treatment regimens, queen sources, and migration patterns) should be incorporated into future surveillance rounds to enable multivariate risk modeling.

## Data Availability

The original contributions presented in the study are included in the article/**Supplementary Material**. Further inquiries can be directed to the corresponding author.

## Supplementary material

Supplementary Figure 1 Pathogen pair co-occurrence by round (diagonal: single-pathogen prevalence; off-diagonal: dual co-occurrence, %). DWV–IAPV co-occurrence intensifies from 15% (R1) to 47% (R3), while DWV–BQCV and DWV–SBV co-occurrence both decline from spring to autumn. KSBV was excluded.
