# Supplementary material for: Seasonal dynamics of honey bee pathogens and Varroa destructor infestation in South Korea: results from a National Active Surveillance Program
Source: Front Insect Sci. 2026 Aug 11;6:1891174. doi: 10.3389/finsc.2026.1891174 (PMC13503378; doi:10.3389/finsc.2026.1891174)
Supplement: Supplementary file 1 [file Supplementaryfile1.docx]

**Supplementary Material**

**Supplementary Figure S1.** Pathogen pair co-occurrence by round (diagonal: single-pathogen prevalence; off-diagonal: dual co-occurrence, %). DWV–IAPV co-occurrence intensifies from 15% (R1) to 47% (R3), while DWV–BQCV and DWV–SBV co-occurrence both decline from spring to autumn. KSBV was excluded.
